# Supplementary material for: A signature invariant geometric algebra framework for spacetime physics and its applications in relativistic dynamics of a massive particle and gyroscopic precession
Source: Sci Rep. 2022 Mar 7;12:3981. doi: 10.1038/s41598-022-06895-0 (PMC8901677; doi:10.1038/s41598-022-06895-0)
Supplement: Supplementary file 1 — Supplementary Information. [file 41598_2022_6895_MOESM1_ESM.pdf]

APPENDIX

APPENDIX A: List of operation rules of blades in the STAs of signatures $(\pm, \mp, \mp, \mp)$

As mentioned in “STAs of signatures $(\pm, \mp, \mp, \mp)$ and their “common” even subalgebra”, once operation rules of blades of different grades in both the STAs are given, one can perform operations between any two multivectors. Based on the general formulas in finite dimensional GA [3, 17], a detail list of operation rules of blades in the two STAs is provided as follows, where since all of the following equations are presented in a signature independent form, the signs “$\pm$” associated with multivectors have been omitted. In the list below, a, b, c, d, e, f, g, and h are vectors, B, B_1, and B_2 are 2-blades, T, T_1, and T_2 are 3-blades, F is 4-blade, and A_r is r-blade $(r = 1, 2, 3, 4)$.

$$aA_r = a \cdot A_r + a \wedge A_r, \ A_r a = A_r \cdot a + A_r \wedge a, \quad (r = 1, 2, 3), \tag{A1}$$

$$B_1 B_2 = B_1 \cdot B_2 + B_1 \times B_2 + B_1 \wedge B_2, \tag{A2}$$

$$BT = B \cdot T + B \times T, \quad TB = T \cdot B + T \times B, \tag{A3}$$

$$T_1 T_2 = T_1 \cdot T_2 + \langle T_1 T_2 \rangle_2, \tag{A4}$$

$$A_r F = A_r \cdot F = (-1)^{3r} F \cdot A_r = (-1)^{3r} F A_r, \quad (r = 1, 2, 3, 4), \tag{A5}$$

$$a \cdot A_r = (-1)^{r-1} A_r \cdot a, \quad a \wedge A_r = (-1)^r A_r \wedge a, \tag{A6}$$

$$a \cdot (b \wedge c \wedge d \wedge \cdots) = (a \cdot b) c \wedge d \wedge \cdots - (a \cdot c) b \wedge d \wedge \cdots + (a \cdot d) b \wedge c \wedge \cdots - \cdots, \tag{A7}$$

$$(a \wedge b) \cdot (c \wedge d) = (c \wedge d) \cdot (a \wedge b) = \det \begin{pmatrix} b \cdot c, & b \cdot d \\ a \cdot c, & a \cdot d \end{pmatrix}, \tag{A8}$$

$$\begin{aligned}(a \wedge b) \cdot (c \wedge d \wedge e) &= (c \wedge d \wedge e) \cdot (a \wedge b) \\ &= \big((a \wedge b) \cdot (c \wedge d)\big) e - \big((a \wedge b) \cdot (c \wedge e)\big) d + \big((a \wedge b) \cdot (d \wedge e)\big) c,\end{aligned} \tag{A9}$$

$$\begin{aligned}(a \wedge b) \cdot (c \wedge d \wedge e \wedge f) &= (c \wedge d \wedge e \wedge f) \cdot (a \wedge b) \\ &= \big((a \wedge b) \cdot (c \wedge d)\big) e \wedge f - \big((a \wedge b) \cdot (c \wedge e)\big) d \wedge f \\ &+ \big((a \wedge b) \cdot (c \wedge f)\big) d \wedge e + \big((a \wedge b) \cdot (d \wedge e)\big) c \wedge f \\ &- \big((a \wedge b) \cdot (d \wedge f)\big) c \wedge e + \big((a \wedge b) \cdot (e \wedge f)\big) c \wedge d,\end{aligned} \tag{A10}$$

$$(a \wedge b \wedge c) \cdot (d \wedge e \wedge f) = (d \wedge e \wedge f) \cdot (a \wedge b \wedge c) = \det \begin{pmatrix} c \cdot d, & c \cdot e, & c \cdot f \\ b \cdot d, & b \cdot e, & b \cdot f \\ a \cdot d, & a \cdot e, & a \cdot f \end{pmatrix}, \tag{A11}$$

$$\begin{aligned}(a \wedge b \wedge c) \cdot (d \wedge e \wedge f \wedge g) &= -(d \wedge e \wedge f \wedge g) \cdot (a \wedge b \wedge c) \\ &= \big((a \wedge b \wedge c) \cdot (d \wedge e \wedge f)\big) g - \big((a \wedge b \wedge c) \cdot (d \wedge e \wedge g)\big) f \\ &+ \big((a \wedge b \wedge c) \cdot (d \wedge f \wedge g)\big) e - \big((a \wedge b \wedge c) \cdot (e \wedge f \wedge g)\big) d,\end{aligned} \tag{A12}$$

$$\begin{aligned}(a \wedge b \wedge c \wedge d) \cdot (e \wedge f \wedge g \wedge h) &= (e \wedge f \wedge g \wedge h) \cdot (a \wedge b \wedge c \wedge d) \\ &= \det \begin{pmatrix} d \cdot e, & d \cdot f, & d \cdot g, & d \cdot h, \\ c \cdot e, & c \cdot f, & c \cdot g, & c \cdot h, \\ b \cdot e, & b \cdot f, & b \cdot g, & b \cdot h, \\ a \cdot e, & a \cdot f, & a \cdot g, & a \cdot h, \end{pmatrix},\end{aligned} \tag{A13}$$

$$B \times A_r = \langle B A_r \rangle_r, \quad A_r \times B = \langle A_r B \rangle_r, \quad (r = 1, 2, 3), \tag{A14}$$

$$\begin{aligned}(a \wedge b) \times (c \wedge d) &= -(c \wedge d) \times (a \wedge b) \\ &= (b \cdot c) a \wedge d - (b \cdot d) a \wedge c + (a \cdot d) b \wedge c - (a \cdot c) b \wedge d,\end{aligned} \tag{A15}$$

$$
\begin{aligned}
(a \wedge b) \times (c \wedge d \wedge e) &= -(c \wedge d \wedge e) \times (a \wedge b) \\
&= (b \cdot c) a \wedge d \wedge e - (b \cdot d) a \wedge c \wedge e + (b \cdot e) a \wedge c \wedge d \\
&\quad - (a \cdot c) b \wedge d \wedge e + (a \cdot d) b \wedge c \wedge e - (a \cdot e) b \wedge c \wedge d, \qquad \text{(A16)} \\
\big\langle (a \wedge b \wedge c)(d \wedge e \wedge f) \big\rangle_2 &= -\big\langle (d \wedge e \wedge f)(a \wedge b \wedge c) \big\rangle_2 \\
&= \big((b \wedge c) \cdot (d \wedge e)\big) a \wedge f - \big((b \wedge c) \cdot (d \wedge f)\big) a \wedge e \\
&\quad + \big((b \wedge c) \cdot (e \wedge f)\big) a \wedge d - \big((a \wedge c) \cdot (d \wedge e)\big) b \wedge f \\
&\quad + \big((a \wedge c) \cdot (d \wedge f)\big) b \wedge e - \big((a \wedge c) \cdot (e \wedge f)\big) b \wedge d \\
&\quad + \big((a \wedge b) \cdot (d \wedge e)\big) c \wedge f - \big((a \wedge b) \cdot (d \wedge f)\big) c \wedge e \\
&\quad + \big((a \wedge b) \cdot (e \wedge f)\big) c \wedge d, \qquad \text{(A17)}
\end{aligned}
$$

where Eqs. (A1)—(A7) and (A14)—(A16) can be directly obtained according to the corresponding formulas in Refs. [3, 17], and the derivations of Eqs. (A8)—(A13) and (A17) are able to be greatly simplified by making use of the bases (2) (cf. (3)) and the anticommutation of the vector generators. In addition, as two typical formulas in GA,

$$a \cdot A_r = \frac{1}{2}\big(aA_r - (-1)^r A_r a\big), \qquad \text{(A18)}$$

$$a \wedge A_r = \frac{1}{2}\big(aA_r + (-1)^r A_r a\big) \qquad \text{(A19)}$$

are also often used in application.

APPENDIX B: The "common" even subalgebra of the STAs of signatures $(\pm, \mp, \mp, \mp)$

In the "common" even subalgebra of the two STAs, all the operation rules are independent of signatures $(\pm, \mp, \mp, \mp)$, and therefore, in this section, the signs "$\pm$" associated with multivectors have been omitted for brevity. According to (25), a basis for this algebraic formalism is

$$\{1, \quad \boldsymbol{\sigma}_k, \quad \boldsymbol{\sigma}_i \boldsymbol{\sigma}_j \ (i < j), \quad \boldsymbol{\sigma}_1 \boldsymbol{\sigma}_2 \boldsymbol{\sigma}_3\}, \qquad \text{(B1)}$$

where $\{\boldsymbol{\sigma}_k\}$, as the vector generators, provide a representation-free version of the Pauli matrices, and from the relevant formulas in "STAs of signatures $(\pm, \mp, \mp, \mp)$ and their "common" even subalgebra", they satisfy the following fundamental properties,

$$
\begin{aligned}
I &= \boldsymbol{\sigma}_1 \boldsymbol{\sigma}_2 \boldsymbol{\sigma}_3, && \text{(B2)} \\
\boldsymbol{\sigma}_i \boldsymbol{\sigma}_j + \boldsymbol{\sigma}_j \boldsymbol{\sigma}_i &= 2\boldsymbol{\sigma}_i \cdot \boldsymbol{\sigma}_j = 2\delta_{ij}, && \text{(B3)} \\
\boldsymbol{\sigma}_i \boldsymbol{\sigma}_j - \boldsymbol{\sigma}_j \boldsymbol{\sigma}_i &= 2\boldsymbol{\sigma}_i \times \boldsymbol{\sigma}_j = 2\epsilon_{ijk} \boldsymbol{\sigma}_k I, && \text{(B4)} \\
\boldsymbol{\sigma}_i \boldsymbol{\sigma}_j &= \delta_{ij} + \epsilon_{ijk} \boldsymbol{\sigma}_k I, && \text{(B5)} \\
\boldsymbol{\sigma}_i \boldsymbol{\sigma}_j &= \boldsymbol{\sigma}_i \times \boldsymbol{\sigma}_j = -\boldsymbol{\sigma}_j \times \boldsymbol{\sigma}_i = -\boldsymbol{\sigma}_i \boldsymbol{\sigma}_j, \quad (i \neq j). && \text{(B6)}
\end{aligned}
$$

Let $\boldsymbol{a} = a_i \boldsymbol{\sigma}_i$, $\boldsymbol{b} = b_j \boldsymbol{\sigma}_j$, and $\boldsymbol{c} = c_k \boldsymbol{\sigma}_k$, and as shown in Eqs. (28)—(30), three types of basic homogeneous multivectors in this algebraic formalism are, respectively, $\boldsymbol{a}$, $\boldsymbol{a} \times \boldsymbol{b}$, and

$$(\boldsymbol{a} \times \boldsymbol{b}) \wedge \boldsymbol{c} = \det \begin{pmatrix} a_1, & b_1, & c_1 \\ a_2, & b_2, & c_2 \\ a_3, & b_3, & c_3 \end{pmatrix} I. \qquad \text{(B7)}$$

By using Eqs. (31)—(39), (21), (A5), and (A9), one gets

$$\boldsymbol{a}\boldsymbol{b} = \boldsymbol{a}\cdot\boldsymbol{b} + \boldsymbol{a}\times\boldsymbol{b}, \tag{B8}$$

$$\boldsymbol{a}\left(\boldsymbol{b}\times\boldsymbol{c}\right) = \boldsymbol{a}\times\left(\boldsymbol{b}\times\boldsymbol{c}\right) + \boldsymbol{a}\wedge\left(\boldsymbol{b}\times\boldsymbol{c}\right), \tag{B9}$$

$$\left(\boldsymbol{b}\times\boldsymbol{c}\right)\boldsymbol{a} = \left(\boldsymbol{b}\times\boldsymbol{c}\right)\times\boldsymbol{a} + \left(\boldsymbol{b}\times\boldsymbol{c}\right)\wedge\boldsymbol{a}, \tag{B10}$$

$$\boldsymbol{a}I = I\boldsymbol{a} = \boldsymbol{a}\cdot I = I\cdot\boldsymbol{a} = \frac{1}{2}\epsilon_{kij}a_k\left(\boldsymbol{\sigma}_i\times\boldsymbol{\sigma}_j\right), \tag{B11}$$

$$\left(\boldsymbol{a}\times\boldsymbol{b}\right)\left(\boldsymbol{c}\times\boldsymbol{d}\right) = \left(\boldsymbol{a}\times\boldsymbol{b}\right)\cdot\left(\boldsymbol{c}\times\boldsymbol{d}\right) + \left(\boldsymbol{a}\times\boldsymbol{b}\right)\times\left(\boldsymbol{c}\times\boldsymbol{d}\right), \tag{B12}$$

$$\left(\boldsymbol{a}\times\boldsymbol{b}\right)I = I\left(\boldsymbol{a}\times\boldsymbol{b}\right) = \left(\boldsymbol{a}\times\boldsymbol{b}\right)\cdot I = I\cdot\left(\boldsymbol{a}\times\boldsymbol{b}\right) = -\epsilon_{kij}a_ib_j\boldsymbol{\sigma}_k, \tag{B13}$$

$$I^2 = II = I\cdot I = -1 \tag{B14}$$

with $\boldsymbol{d} = d_p\boldsymbol{\sigma}_p$,

$$\boldsymbol{a}\times\left(\boldsymbol{b}\times\boldsymbol{c}\right) = -\left(\boldsymbol{b}\times\boldsymbol{c}\right)\times\boldsymbol{a} = \left(\boldsymbol{a}\cdot\boldsymbol{b}\right)\boldsymbol{c} - \left(\boldsymbol{a}\cdot\boldsymbol{c}\right)\boldsymbol{b}, \tag{B15}$$

$$\boldsymbol{a}\wedge\left(\boldsymbol{b}\times\boldsymbol{c}\right) = \left(\boldsymbol{b}\times\boldsymbol{c}\right)\wedge\boldsymbol{a} = \boldsymbol{b}\wedge\left(\boldsymbol{c}\times\boldsymbol{a}\right) = \boldsymbol{c}\wedge\left(\boldsymbol{a}\times\boldsymbol{b}\right), \tag{B16}$$

$$\left(\boldsymbol{a}\times\boldsymbol{b}\right)\cdot\left(\boldsymbol{c}\times\boldsymbol{d}\right) = \left(\boldsymbol{b}\cdot\boldsymbol{c}\right)\left(\boldsymbol{a}\cdot\boldsymbol{d}\right) - \left(\boldsymbol{b}\cdot\boldsymbol{d}\right)\left(\boldsymbol{a}\cdot\boldsymbol{c}\right), \tag{B17}$$

$$\begin{aligned}\left(\boldsymbol{a}\times\boldsymbol{b}\right)\times\left(\boldsymbol{c}\times\boldsymbol{d}\right) &= \left(\boldsymbol{b}\cdot\boldsymbol{c}\right)\left(\boldsymbol{a}\times\boldsymbol{d}\right) + \left(\boldsymbol{a}\cdot\boldsymbol{d}\right)\left(\boldsymbol{b}\times\boldsymbol{c}\right)\\ &\quad - \left(\boldsymbol{b}\cdot\boldsymbol{d}\right)\left(\boldsymbol{a}\times\boldsymbol{c}\right) - \left(\boldsymbol{a}\cdot\boldsymbol{c}\right)\left(\boldsymbol{b}\times\boldsymbol{d}\right).\end{aligned} \tag{B18}$$

These formulas constitute the main operation rules of geometric product, inner product, wedge product, and commutator product in the "common" even subalgebra of the two STAs.

As noted in "STAs of signatures $(\pm,\mp,\mp,\mp)$ and their "common" even subalgebra", the relative space spanned by $\{\boldsymbol{\sigma}_k\}$ is an Euclidean space of dimension 3 with I as a pseudoscalar, and in this space, the inner product and the cross product between two relative vectors are well-defined. From Eqs. (18) and (19), the inner product and the cross product between relative vectors $\boldsymbol{a}$ and $\boldsymbol{b}$ are

$$\boldsymbol{a}\cdot\boldsymbol{b} = \langle\boldsymbol{a}\boldsymbol{b}\rangle = a_kb_k, \tag{B19}$$

$$\boldsymbol{a}\times_3\boldsymbol{b} = -I\left(\boldsymbol{a}\times\boldsymbol{b}\right) = \epsilon_{ijk}a_ib_j\boldsymbol{\sigma}_k, \tag{B20}$$

which are identical to their conventional ones, respectively. Eq. (B20) suggests that in the relative space, $\boldsymbol{a}\times_3\boldsymbol{b}$ is actually the dual of $\boldsymbol{a}\times\boldsymbol{b}$, and with this result, the commutator products in Eqs. (B15)—(B18) can be replaced by the corresponding cross products. By applying Eqs. (B20), (B13), and (B14), the equalities

$$\begin{aligned}\boldsymbol{a}\times_3\left(\boldsymbol{b}\times_3\boldsymbol{c}\right) &= \{\boldsymbol{a}\times[\left(\boldsymbol{b}\times\boldsymbol{c}\right)I]\}I = \langle\boldsymbol{a}\left(\boldsymbol{b}\times\boldsymbol{c}\right)I\rangle_2 I = \langle\boldsymbol{a}\left(\boldsymbol{b}\times\boldsymbol{c}\right)\rangle_2 I^2\\ &= -\boldsymbol{a}\times\left(\boldsymbol{b}\times\boldsymbol{c}\right),\end{aligned} \tag{B21}$$

$$\begin{aligned}\boldsymbol{a}\cdot\left(\boldsymbol{b}\times_3\boldsymbol{c}\right)I &= -\{\boldsymbol{a}\cdot[\left(\boldsymbol{b}\times\boldsymbol{c}\right)I]\}I = -\langle\boldsymbol{a}\left(\boldsymbol{b}\times\boldsymbol{c}\right)I\rangle I = -\langle\boldsymbol{a}\left(\boldsymbol{b}\times\boldsymbol{c}\right)\rangle_4 I^2\\ &= \boldsymbol{a}\wedge\left(\boldsymbol{b}\times\boldsymbol{c}\right),\end{aligned} \tag{B22}$$

$$\begin{aligned}\left(\boldsymbol{a}\times_3\boldsymbol{b}\right)\cdot\left(\boldsymbol{c}\times_3\boldsymbol{d}\right) &= \langle\left(\boldsymbol{a}\times\boldsymbol{b}\right)I\left(\boldsymbol{c}\times\boldsymbol{d}\right)I\rangle = \langle\left(\boldsymbol{a}\times\boldsymbol{b}\right)\left(\boldsymbol{c}\times\boldsymbol{d}\right)I^2\rangle\\ &= -\langle\left(\boldsymbol{a}\times\boldsymbol{b}\right)\left(\boldsymbol{c}\times\boldsymbol{d}\right)\rangle = -\left(\boldsymbol{a}\times\boldsymbol{b}\right)\cdot\left(\boldsymbol{c}\times\boldsymbol{d}\right),\end{aligned} \tag{B23}$$

$$\begin{aligned}\left(\boldsymbol{a}\times_3\boldsymbol{b}\right)\times_3\left(\boldsymbol{c}\times_3\boldsymbol{d}\right) &= -\langle\left(\boldsymbol{a}\times\boldsymbol{b}\right)I\left(\boldsymbol{c}\times\boldsymbol{d}\right)I\rangle_2 I = -\langle\left(\boldsymbol{a}\times\boldsymbol{b}\right)\left(\boldsymbol{c}\times\boldsymbol{d}\right)I^2\rangle_2 I\\ &= \langle\left(\boldsymbol{a}\times\boldsymbol{b}\right)\left(\boldsymbol{c}\times\boldsymbol{d}\right)\rangle_2 I = \left(\left(\boldsymbol{a}\times\boldsymbol{b}\right)\times\left(\boldsymbol{c}\times\boldsymbol{d}\right)\right)I\end{aligned} \tag{B24}$$

are deduced, and then, plugging them into Eqs. (B15)—(B18) gives

$$\boldsymbol{a}\times_3\left(\boldsymbol{b}\times_3\boldsymbol{c}\right) = \left(\boldsymbol{a}\cdot\boldsymbol{c}\right)\boldsymbol{b} - \left(\boldsymbol{a}\cdot\boldsymbol{b}\right)\boldsymbol{c}, \tag{B25}$$

$$\boldsymbol{a}\cdot\left(\boldsymbol{b}\times_3\boldsymbol{c}\right) = \boldsymbol{b}\cdot\left(\boldsymbol{c}\times_3\boldsymbol{a}\right) = \boldsymbol{c}\cdot\left(\boldsymbol{a}\times_3\boldsymbol{b}\right) = \det\begin{pmatrix}a_1, & b_1, & c_1\\ a_2, & b_2, & c_2\\ a_3, & b_3, & c_3\end{pmatrix}, \tag{B26}$$

$$\left(\boldsymbol{a}\times_3\boldsymbol{b}\right)\cdot\left(\boldsymbol{c}\times_3\boldsymbol{d}\right) = \left(\boldsymbol{a}\cdot\boldsymbol{c}\right)\left(\boldsymbol{b}\cdot\boldsymbol{d}\right) - \left(\boldsymbol{a}\cdot\boldsymbol{d}\right)\left(\boldsymbol{b}\cdot\boldsymbol{c}\right), \tag{B27}$$

$$\begin{aligned}\left(\boldsymbol{a}\times_3\boldsymbol{b}\right)\times_3\left(\boldsymbol{c}\times_3\boldsymbol{d}\right) &= \left(\boldsymbol{a}\cdot\boldsymbol{c}\right)\left(\boldsymbol{b}\times_3\boldsymbol{d}\right) + \left(\boldsymbol{b}\cdot\boldsymbol{d}\right)\left(\boldsymbol{a}\times_3\boldsymbol{c}\right)\\ &\quad - \left(\boldsymbol{a}\cdot\boldsymbol{d}\right)\left(\boldsymbol{b}\times_3\boldsymbol{c}\right) - \left(\boldsymbol{b}\cdot\boldsymbol{c}\right)\left(\boldsymbol{a}\times_3\boldsymbol{d}\right).\end{aligned} \tag{B28}$$

Eqs. (B25)—(B28) are exactly those formulas involving cross product in vector analysis, which implies that the relative space, as an Euclidean space of dimension 3, can be treated as an arena for classical physics.

Functions defined on finite dimensional GA have a wide range of applications in physics, and readers interested in the details on this topics are invited to consult Refs. [4–7, 17]. Here, we only focus on those defined on the "common" even subalgebra of the STAs of signatures $(\pm, \mp, \mp, \mp)$. The exponential function is the most common one, and for a multivector A, its exponential function is defined by

$$\mathrm{e}^{A} = \sum_{m=0}^{\infty} \frac{A^{\langle m \rangle}}{m!} \tag{B29}$$

with

$$A^{\langle m \rangle} := \begin{cases} (A \cdot A)^{\frac{m}{2}}, & \text{for even } m, \\ (A \cdot A)^{\frac{m-1}{2}} A, & \text{for odd } m, \end{cases} \tag{B30}$$

where the series is absolutely convergent [5–7]. Starting from the equality $A \cdot A = \tilde{A} \cdot \tilde{A}$ [3], one property of e^{A} can be directly obtained,

$$\widetilde{\mathrm{e}^{A}} = \mathrm{e}^{\tilde{A}}. \tag{B31}$$

Note that $A^{\langle m \rangle}$ is not equivalent to $A^{m} := AA \cdots A$ in the general case. The odd and even parts of the exponential function are referred to as the hyperbolic sine and cosine functions, respectively,

$$\begin{cases} \sinh A := \sum_{n=0}^{\infty} \frac{A^{\langle 2n+1 \rangle}}{(2n+1)!} = \frac{\mathrm{e}^{A} - \mathrm{e}^{-A}}{2}, \\ \cosh A := \sum_{n=0}^{\infty} \frac{A^{\langle 2n \rangle}}{(2n)!} = \frac{\mathrm{e}^{A} + \mathrm{e}^{-A}}{2}, \end{cases} \tag{B32}$$

and thus,

$$\mathrm{e}^{A} = \cosh A + \sinh A. \tag{B33}$$

Similarly, the trigonometric functions sine and cosine are also defined by power series in the normal way,

$$\begin{cases} \sin A = \sum_{n=0}^{\infty} (-1)^{n} \frac{A^{\langle 2n+1 \rangle}}{(2n+1)!}, \\ \cos A = \sum_{n=0}^{\infty} (-1)^{n} \frac{A^{\langle 2n \rangle}}{(2n)!}. \end{cases} \tag{B34}$$

We are now in a position to find the relationship between hyperbolic and trigonometric functions. Let J be a multivector satisfying $|J^{\langle 2 \rangle}| = 1$ and $JA = AJ$, where $|J^{\langle 2 \rangle}|$ denotes the absolute value of $J^{\langle 2 \rangle}$. By inserting JA into Eqs. (B32) and (B34), the relationships of the functions sin, cos, sinh, and cosh are derived,

$$\sinh(JA) = J \sinh A, \quad \text{for} \quad J^{\langle 2 \rangle} = 1, \tag{B35a}$$
$$\sinh(JA) = J \sin A, \quad \text{for} \quad J^{\langle 2 \rangle} = -1, \tag{B35b}$$
$$\cosh(JA) = \cosh A, \quad \text{for} \quad J^{\langle 2 \rangle} = 1, \tag{B35c}$$
$$\cosh(JA) = \cos A, \quad \text{for} \quad J^{\langle 2 \rangle} = -1 \tag{B35d}$$

and

$$\sin(JA) = J \sin A, \quad \text{for} \quad J^{\langle 2 \rangle} = 1, \tag{B36a}$$
$$\sin(JA) = J \sinh A, \quad \text{for} \quad J^{\langle 2 \rangle} = -1, \tag{B36b}$$
$$\cos(JA) = \cos A, \quad \text{for} \quad J^{\langle 2 \rangle} = 1, \tag{B36c}$$
$$\cos(JA) = \cosh A, \quad \text{for} \quad J^{\langle 2 \rangle} = -1. \tag{B36d}$$

Based on these equalities, with Eq. (B33), we arrive at

$$\mathrm{e}^{JA} = \begin{cases} \cosh A + J \sinh A, & \text{for} \quad J^{\langle 2 \rangle} = 1, \\ \cos A + J \sin A, & \text{for} \quad J^{\langle 2 \rangle} = -1. \end{cases} \tag{B37}$$

Next, starting from Eq. (B37), a simple method to construct rotors will be provided. Let α and B be a real number and a unit 2-blade, respectively, and we shall prove that $\mathrm{e}^{\alpha B}$ is a rotor. Here, as a unit 2-blade, B is defined by

$$B^2 = 1 \quad \text{or} \quad B^2 = -1, \tag{B38}$$

and one needs to note that in such a case, $B^2 = B^{\langle 2 \rangle} = B \cdot B$ holds. Thus, by substitution of Eq. (B37), $\mathrm{e}^{\alpha B}$ can be written as

$$\mathrm{e}^{\alpha B} = \begin{cases} \cosh \alpha + B \sinh \alpha, & \text{for} \quad B^2 = 1, \\ \cos \alpha + B \sin \alpha, & \text{for} \quad B^2 = -1, \end{cases} \tag{B39}$$

and applying this result repeatedly, we find that

$$\mathrm{e}^{\alpha_1 B} \mathrm{e}^{\alpha_2 B} = \mathrm{e}^{(\alpha_1 + \alpha_2) B} \tag{B40}$$

with α_1 and α_2 as arbitrary real numbers, where the particular case,

$$\mathrm{e}^{\alpha B} \mathrm{e}^{-\alpha B} = 1, \tag{B41}$$

is also worth noting. Besides, with the help of Eq. (B31), the above equation is equivalent to

$$\mathrm{e}^{\alpha B} \widetilde{\mathrm{e}^{\alpha B}} = 1. \tag{B42}$$

Eqs. (B39) and (B42) clearly suggest that $\mathrm{e}^{\alpha B}$ is an even multivector satisfying $\mathrm{e}^{\alpha B} \widetilde{\mathrm{e}^{\alpha B}} = 1$. Then, according to the definition of rotor (cf. "Rotor techniques on Lorentz boost and spatial rotation"), one only needs to prove that in spacetime, the map defined by $b \mapsto \mathrm{e}^{\alpha B} b \, \widetilde{\mathrm{e}^{\alpha B}}$ transforms any vector into another one. For an arbitrary vector b, Eq. (A1) provides the decomposition,

$$b = (b \cdot B) B^{-1} + (b \wedge B) B^{-1}. \tag{B43}$$

Since Eq. (B38) indicates that

$$B^{-1} = B \quad \text{or} \quad B^{-1} = -B, \tag{B44}$$

one can directly verify that

$$(b \cdot B) \wedge B^{-1} = 0, \tag{B45a}$$

$$(b \wedge B) \times B^{-1} = \langle b B B^{-1} \rangle_3 - (b \cdot B) \wedge B^{-1} = 0. \tag{B45b}$$

Thus, by using Eqs. (A1) and (A3), the following results hold:

$$(b \cdot B) B^{-1} = (b \cdot B) \cdot B^{-1} =: b_{\|}, \tag{B46a}$$

$$(b \wedge B) B^{-1} = (b \wedge B) \cdot B^{-1} =: b_{\perp} \tag{B46b}$$

with

$$b = b_{\|} + b_{\perp} \tag{B47}$$

and

$$b_{\|} \wedge B = \langle (b \cdot B) B^{-1} B \rangle_3 = 0, \tag{B48a}$$

$$b_{\perp} \cdot B = \langle (b \wedge B) B^{-1} B \rangle_1 = 0, \tag{B48b}$$

where $b_{\|}$ and $b_{\perp}$ are obviously the components of b parallel and perpendicular to B, respectively. Furthermore, by virtue of Eqs. (A1) and (A6), two important equalites

$$b_{\|}B = b_{\|}\cdot B = -B\cdot b_{\|} = -Bb_{\|}, \tag{B49a}$$

$$b_{\perp}B = b_{\perp}\wedge B = B\wedge b_{\perp} = Bb_{\perp} \tag{B49b}$$

are obtained, and then, together with Eqs. (B47), (B31), (B39), and (B40), we finally get

$$\mathrm{e}^{\alpha B}b\,\widetilde{\mathrm{e}^{\alpha B}} = \mathrm{e}^{\alpha B}\left(b_{\|}+b_{\perp}\right)\mathrm{e}^{-\alpha B} = \mathrm{e}^{2\alpha B}b_{\|}+b_{\perp} = \cos\left(2\alpha\right)b_{\|}-\sin\left(2\alpha\right)b_{\|}\cdot B+b_{\perp}. \tag{B50}$$

Evidently, $\mathrm{e}^{\alpha B}b\,\widetilde{\mathrm{e}^{\alpha B}}$ is a vector, and therefore, the map defined by $b\mapsto \mathrm{e}^{\alpha B}b\,\widetilde{\mathrm{e}^{\alpha B}}$ is indeed a transformation in spacetime. In the STAs of signatures $(\pm,\mp,\mp,\mp)$, the rotor $\mathrm{e}^{\alpha B}$ constructed above can be employed to handle Lorentz boost and spatial rotation, and we will discuss this topic in "Rotor techniques on Lorentz boost and spatial rotation".

APPENDIX C: A local orthonormal tetrad $\{\gamma_\alpha\}$ and the bivector connection $\omega(u)$ associated with it in the Lense-Thirring spacetime

The Lense-Thirring metric in isotropic coordinates has the form of the $1/c$ expansion [30],

$$\begin{cases} g_{00} = \pm\left(1-\dfrac{2}{c^2}U\right), \\ g_{0i} = \pm\dfrac{4}{c^3}V_i, \\ g_{ij} = \mp\delta_{ij}\left(1+\dfrac{2}{c^2}U\right), \end{cases} \tag{C1}$$

where the potentials U and V_i are defined in Eq. (126), and by applying Eqs. (A8), (A11), and (A13), the following quantities expanded up to $1/c^3$ order are given,

$$(g_1\wedge g_0)\cdot(g_0\wedge g_1) = -1, \tag{C2a}$$

$$(g_1\wedge g_0)\cdot(g_0\wedge g_2) = 0, \tag{C2b}$$

$$(g_1\wedge g_0)\cdot(g_0\wedge g_3) = 0, \tag{C2c}$$

$$(g_1\wedge g_0)\cdot(g_1\wedge g_2) = \frac{4}{c^3}V_2, \tag{C2d}$$

$$(g_2\wedge g_1\wedge g_0)\cdot(g_0\wedge g_1\wedge g_2) = \pm\left(1+\frac{2}{c^2}U\right), \tag{C3a}$$

$$(g_2\wedge g_1\wedge g_0)\cdot(g_0\wedge g_1\wedge g_3) = 0, \tag{C3b}$$

$$(g_2\wedge g_1\wedge g_0)\cdot(g_0\wedge g_2\wedge g_3) = 0, \tag{C3c}$$

$$(g_2\wedge g_1\wedge g_0)\cdot(g_1\wedge g_2\wedge g_3) = \pm\frac{4}{c^3}V_3, \tag{C3d}$$

and

$$(g_3\wedge g_2\wedge g_1\wedge g_0)\cdot(g_0\wedge g_1\wedge g_2\wedge g_3) = -\left(1+\frac{4}{c^2}U\right). \tag{C4}$$

Eqs. (C1), (C2a), (C3a), and (C4) show that Eqs. (78a)—(78d) hold, which implies that we are capable of assuming that there exists a collection of fiducial observers who are distributed over space and at rest in the coordinate system of $g_{\mu\nu}$. As a result, with the help of the relevant formulas in Appendix A, by inserting Eqs. (77) and (C2a)—(C4) into Eq. (79), a local orthonormal tetrad $\{\gamma_\alpha\}$ determined up to $1/c^3$ order in the Lense-Thirring spacetime is acquired,

$$\begin{cases} \gamma_0 = \left(1+\dfrac{1}{c^2}U\right)g_0, \\ \gamma_i = -\dfrac{4}{c^3}V_i g_0+\left(1-\dfrac{1}{c^2}U\right)g_i, \end{cases}$$

namely Eq. (125).

Next, the bivector connection $\omega(u)$ associated with $\{\gamma_\alpha\}$ will be derived, and the relevant computations are greatly simplified by the condition "up to $1/c^3$ order". Plugging Eqs. (77) and (C1)—(C4) into Eq. (88) yields

$$\begin{cases} g_0 = \left(1 - \frac{1}{c^2}U\right)\gamma_0, \\ g_i = \frac{4}{c^3}V_i\gamma_0 + \left(1 + \frac{1}{c^2}U\right)\gamma_i, \end{cases} \tag{C5}$$

and with them, one is able to deduce

$$\begin{cases} g_0 \wedge g_1 \wedge g_2 = \left(1 + \frac{1}{c^2}U\right)\gamma_0\gamma_1\gamma_2, \\ g_0 \wedge g_1 \wedge g_3 = \left(1 + \frac{1}{c^2}U\right)\gamma_0\gamma_1\gamma_3, \\ g_0 \wedge g_2 \wedge g_3 = \left(1 + \frac{1}{c^2}U\right)\gamma_0\gamma_2\gamma_3, \\ g_1 \wedge g_2 \wedge g_3 = \frac{4}{c^3}V_1\gamma_0\gamma_2\gamma_3 - \frac{4}{c^3}V_2\gamma_0\gamma_1\gamma_3 + \frac{4}{c^3}V_3\gamma_0\gamma_1\gamma_2 + \left(1 + \frac{3}{c^2}U\right)\gamma_1\gamma_2\gamma_3, \\ g_0 \wedge g_1 \wedge g_2 \wedge g_3 = \left(1 + \frac{2}{c^2}U\right)\gamma_0\gamma_1\gamma_2\gamma_3, \end{cases} \tag{C6}$$

where the orthogonality and the anticommutation of $\{\gamma_\alpha\}$ are used. Thus, let $\{\gamma^\beta\}$ be the reciprocal tetrad of $\{\gamma_\alpha\}$, and then, the reciprocal frame $\{g^\mu\}$ of the coordinate frame can be constructed by Eq. (87),

$$\begin{cases} g^0 = \left(1 + \frac{1}{c^2}U\right)\gamma^0 - \frac{4}{c^3}V_j\gamma^j, \\ g^i = \left(1 - \frac{1}{c^2}U\right)\gamma^i, \end{cases} \tag{C7}$$

with which, one further obtains

$$\begin{cases} g^0 \wedge g^j = \gamma^0 \wedge \gamma^j - \frac{4}{c^3}V_k\gamma^k \wedge \gamma^j, \\ g^i \wedge g^j = \left(1 - \frac{2}{c^2}U\right)\gamma^i \wedge \gamma^j. \end{cases} \tag{C8}$$

Besides, Eqs. (C1) and (C5) provide

$$\begin{cases} \partial_0 g_{00} = \partial_0 g_{0i} = 0, \\ \partial_j g_{00} = \mp\frac{2}{c^2}\partial_j U, \\ \partial_j g_{0i} = \pm\frac{4}{c^3}\partial_j V_i, \\ \partial_0 g_{k0} = \partial_0 g_{ki} = 0, \\ \partial_j g_{k0} = \pm\frac{4}{c^3}\partial_j V_k, \\ \partial_j g_{ki} = \mp\frac{2}{c^2}\partial_j U\delta_{ki} \end{cases} \tag{C9}$$

and

$$\begin{cases} g_0 \cdot \partial g_0 = g_0 \cdot \partial g_i = 0, \\ g_j \cdot \partial g_0 = -\frac{1}{c^2}\partial_j U\gamma_0 \\ g_j \cdot \partial g_i = \frac{4}{c^3}\partial_j V_i\gamma_0 + \frac{1}{c^2}\partial_j U\gamma_i, \end{cases} \tag{C10}$$

respectively, where in the derivation of Eq. (C10), Eq. (83a) has been employed. The substitution of Eqs. (C8)—(C10) in Eq. (86) gives rise to the connection bivectors $\omega(g_0)$ and $\omega(g_k)$ expanded up to $1/c^3$ order,

$$\begin{cases} \omega(g_0) = -\frac{1}{c^2}\partial_j U \boldsymbol{\sigma}^j - \frac{2}{c^3}\partial_j V_k \boldsymbol{\sigma}^k \times \boldsymbol{\sigma}^j, \\ \omega(g_i) = \frac{1}{c^2}\partial_j U \boldsymbol{\sigma}^i \times \boldsymbol{\sigma}^j + \frac{2}{c^3}\partial_j V_i \boldsymbol{\sigma}^j - \frac{2}{c^3}\partial_i V_j \boldsymbol{\sigma}^j, \end{cases} \tag{C11}$$

in which, $\{\boldsymbol{\sigma}^k := \gamma_0\gamma^k = \pm\gamma^0\gamma^k\}$ is the reciprocal frame of $\{\boldsymbol{\sigma}_k\}$, and as in Eq. (8), there is $\boldsymbol{\sigma}^i \times \boldsymbol{\sigma}^j = \mp\gamma^i \wedge \gamma^j$. Together with Eqs. (125), (128), and (130), the four-velocity u of the gyroscope can also be expanded in the coordinate frame $\{g_\mu\}$,

$$\begin{aligned} u &= \left(1 + \frac{1}{2c^2}\boldsymbol{u}^2\right)\left[c\left(1 + \frac{1}{c^2}U\right)g_0 + u^i\left(-\frac{4}{c^3}V_i g_0 + \left(1 - \frac{1}{c^2}U\right)g_i\right)\right] \\ &= \left[c\left(1 + \frac{1}{2c^2}\boldsymbol{u}^2 + \frac{1}{c^2}U\right) - \frac{4}{c^3}u^i V_i\right]g_0 + \left(1 + \frac{1}{2c^2}\boldsymbol{u}^2 - \frac{1}{c^2}U\right)u^i g_i, \end{aligned} \tag{C12}$$

and then, by applying Eq. (85), the expression of the bivector connection $\omega(u)$ associated with $\{\gamma_\alpha\}$ up to $1/c^3$ order is achieved,

$$\begin{aligned} \omega(u) &= c\left(1 + \frac{1}{2c^2}\boldsymbol{u}^2 + \frac{1}{c^2}U\right)\omega(g_0) + \left(1 + \frac{1}{2c^2}\boldsymbol{u}^2 - \frac{1}{c^2}U\right)u^i\omega(g_i) \\ &= -\frac{1}{c}\partial_j U\boldsymbol{\sigma}^j - \frac{2}{c^2}\partial_j V_k\boldsymbol{\sigma}^k \times \boldsymbol{\sigma}^j - \frac{1}{2c^3}\boldsymbol{u}^2\partial_j U\boldsymbol{\sigma}^j - \frac{1}{c^3}U\partial_j U\boldsymbol{\sigma}^j \\ &\quad + \frac{1}{c^2}u^i\partial_j U\boldsymbol{\sigma}^i \times \boldsymbol{\sigma}^j + \frac{2}{c^3}u^i\left(\partial_j V_i - \partial_i V_j\right)\boldsymbol{\sigma}^j \\ &= -\frac{1}{c}\boldsymbol{\nabla}U + \frac{2}{c^2}\boldsymbol{\nabla} \times \boldsymbol{V} - \frac{1}{2c^3}\boldsymbol{u}^2\boldsymbol{\nabla}U - \frac{1}{c^3}U\boldsymbol{\nabla}U + \frac{1}{c^2}\boldsymbol{u} \times \boldsymbol{\nabla}U - \frac{2}{c^3}\boldsymbol{u} \times (\boldsymbol{\nabla} \times \boldsymbol{V}) \end{aligned} \tag{C13}$$

with $\boldsymbol{\nabla} := \boldsymbol{\sigma}^k\partial_k$ and $\boldsymbol{V} := V_i\boldsymbol{\sigma}_i$. Finally, according to Eqs. (100a)—(100c), the corresponding expressions of the electric part $\omega^{(E)}(u)$ and the magnetic part $\omega^{(B)}(u)$ of $\omega(u)$ are, respectively, evaluated as

$$\omega^{(E)}(u) = -\frac{1}{c}\boldsymbol{\nabla}U - \frac{1}{2c^3}\boldsymbol{u}^2\boldsymbol{\nabla}U - \frac{1}{c^3}U\boldsymbol{\nabla}U - \frac{2}{c^3}\boldsymbol{u} \times (\boldsymbol{\nabla} \times \boldsymbol{V}), \tag{C14}$$

$$\omega^{(B)}(u) = \frac{2}{c^2}\boldsymbol{\nabla} \times \boldsymbol{V} + \frac{1}{c^2}\boldsymbol{u} \times \boldsymbol{\nabla}U. \tag{C15}$$
